# Burden of tension-type headache in the Middle East and North Africa region, 1990-2019

**DOI:** 10.1186/s10194-022-01445-5

**Published:** 2022-07-06

**Authors:** Saeid Safiri, Ali-Asghar Kolahi, Maryam Noori, Seyed Aria Nejadghaderi, Armin Aslani, Mark J. M. Sullman, Mehdi Farhoudi, Mostafa Araj-Khodaei, Gary S. Collins, Jay S. Kaufman, Kurosh Gharagozli

**Affiliations:** 1 Neurosciences Research Center, Aging Research Institute, Tabriz University of Medical Sciences, Tabriz, Iran; 2 Social Determinants of Health Research Center, Department of Community Medicine, Faculty of Medicine, Tabriz University of Medical Sciences, Tabriz, Iran; 3 Social Determinants of Health Research Center, Shahid Beheshti University of Medical Sciences, Tehran, Iran; 4 Student Research Committee, School of Medicine, Iran University of Medical Sciences, Tehran, Iran; 5 Urology Research Center, Tehran University of Medical Sciences, Tehran, Iran; 6 Research Center for Integrative Medicine in Aging, Aging Research Institute, Tabriz University of Medical Sciences, Tabriz, Iran; 7 Systematic Review and Meta-analysis Expert Group (SRMEG), Universal Scientific Education and Research Network (USERN), Tehran, Iran; 8 Department of Life and Health Sciences, University of Nicosia, Nicosia, Cyprus; 9 Department of Social Sciences, University of Nicosia, Nicosia, Cyprus; 10 Centre for Statistics in Medicine, NDORMS, Botnar Research Centre, University of Oxford, Oxford, UK; 11 NIHR Oxford Biomedical Research Centre, Oxford University Hospitals NHS Foundation Trust, Oxford, UK; 12 Department of Epidemiology, Biostatistics and Occupational Health, Faculty of Medicine, McGill University, Montreal, Quebec Canada; 13 Brain Mapping Research Center, Shahid Beheshti University of Medical Sciences, Tehran, Iran

**Keywords:** Headache, Epidemiology, Incidence, Prevalence, Risk Factors, Middle East and North Africa, Eastern Mediterranean

## Abstract

**Introduction:**

Tension-type headache (TTH) is the most prevalent neurological disorder. As there is a gap in the literature regarding the disease burden attributable to TTH in the Middle East and North Africa (MENA) region, the aim of the present study was to report the epidemiological indicators of TTH in MENA, from 1990 to 2019, by sex, age and socio-demographic index (SDI).

**Methods:**

Publicly available data on the point prevalence, annual incidence and years lived with disability (YLDs) were retrieved from the global burden of disease (GBD) 2019 study for the 21 countries and territories in MENA, between 1990 and 2019. The results were presented with numbers and age-standardised rates per 100000 population, along with their corresponding 95% uncertainty intervals (UIs).

**Results:**

In 2019, the age-standardised point prevalence and annual incidence rates for TTH in the MENA region were 24504.5 and 8680.1 per 100000, respectively, which represents a 2.0% and a 0.9% increase over 1990-2019, respectively. The age-standardised YLD rate of TTH in this region in 2019 was estimated to be 68.1 per 100000 population, which has increased 1.0% since 1990. Iran [29640.4] had the highest age-standardised point prevalence rate for TTH, while Turkey [21726.3] had the lowest. In 2019, the regional point prevalence of TTH was highest in the 35-39 and 70-74 age groups, for males and females, respectively. Furthermore, the number of prevalent cases was estimated to be highest in those aged 35-39 and 25-29 years, in both males and females, respectively. Moreover, the burden of TTH was not observed to have a clear association with SDI.

**Conclusions:**

While the prevalence of TTH in the MENA region increased from 1990 to 2019, the incidence rate did not change. In addition, the burden of TTH in MENA was higher than at the global level for both sexes and all age groups. Therefore, prevention of TTH would help alleviate the attributable burden imposed on the hundreds of millions of people suffering from TTH around the region.

**Supplementary Information:**

The online version contains supplementary material available at 10.1186/s10194-022-01445-5.

## Introduction

Tension-type headache (TTH) is the most prevalent neurological disorder [1]. TTH is characterised by mild to moderate bilateral pain in the temporal area, often described as band-like pain according to the International Classification of Headache Disorders (ICHD) version 3 [2, 3]. Fatigue, stress, anxiety, temperature, sleep deprivation and specific foods may aggravate the condition, while sleep, medications, rest, posture and massage can help to relieve the pain [4]. Stress is the most common trigger, but depression, anxiety and muscle tension may also be crucial risk factors for TTHs [4, 5]. TTH is distinguished from migraine by the absence of nausea, photophobia, phonophobia and other neurological symptoms [6].

According to the Global Burden of Disease (GBD) study 2016, about 1.89 billion people suffer from TTH [7]. This number reached 2.3 billion people in 2017 [8, 9], while the incidence of TTH was estimated to be 11.4 thousand per 100,000 [8, 9]. Globally, TTHs caused 7.2 million years lived with disability (YLD) [7]. Furthermore, based on the pooled results from five population-based studies, the mean lifetime prevalence of TTHs in adults is 46% (range 12-78%) [10]. Although children may also be affected, prevalence peaks in those aged 40-49 years for both sexes. The female to male ratio of TTH is about 5:4 and this ratio widens as headaches become more chronic [11]. The burden of TTH increased slightly by raising the developmental status of the countries at the global level and the Middle East and North Africa (MENA) region was among the regions that had higher than expected burden [7]. According to the GBD 2016, more than 175 million people in the MENA region had TTHs, and the mean burden of this disease was more than 777 thousand YLDs [7].

A number of studies have reported the prevalence of this disorder at the global level, but these studies do not provide detailed information regarding each individual region or the countries within these regions. The lack of granularity can be problematic, since regional patterns may vary substantially from global patterns, and using information on the global situation might be misleading for healthcare policymaking in the individual countries. Furthermore, the latest GBD study was conducted in 2016 and is now out of date [7]. Although epidemiological studies should be regularly updated, and despite the importance of headache disorders for public health, there has been no comprehensive study investigating the burden of TTH within the MENA region. Therefore, using data from the GBD study 2019, the present study reported the point prevalence, annual incidence and YLDs of TTH in the MENA region from 1990 to 2019, by sex, age and socio-demographic index (SDI).

## Methods

### Overview

GBD 2019 measured the levels and trends associated with 369 diseases and injuries and 87 risk factors, from 1990 to 2019, in 204 countries and territories, 7 super-regions and 21 regions [12]. A detailed description of the methodology used for estimating the burden of diseases in GBD 2019 has been previously reported [8, 12, 13] and the data can be found here: https://vizhub.healthdata.org/gbd-compare/ and http://ghdx.healthdata.org/gbd-results-tool.

### Case definition and data sources

TTH is defined as a dull, diffuse, non-pulsatile, band-like (or vice-like) pain which is mild to moderate in intensity and is located in the head and/or neck. For the present study, the ICHD (version 3) criteria was used as the definition of TTH [3]. When a headache satisfies all domains of the criteria it is called a definite TTH, while a headache which satisfies all but one is labelled probable TTH. Prior to GBD 2017, no distinction was made between probable and definite TTH. However, since this time a number of different case definitions have been used and these were also included [14].

The most recent systematic review of TTH was undertaken for GBD 2017, and included articles published up to the end of September 2017 [14]. The search strings used during this review were ((((("headache"[MeSH Terms]) OR ("headache"[Title/Abstract] AND "tension"[Title/Abstract])) AND ("epidemiology"[Title/Abstract] OR "prevalence"[Title/Abstract] OR "incidence"[Title/Abstract] OR "remission"[Title/Abstract])))). Studies were excluded that were not representative of the population or did not report the prevalence of TTH. Furthermore, the adjustments required to make medical claims data compatible with the representative survey data were unsound and so medical claims data were also excluded. A detailed description of the data sources used to model TTH can be found here: https://ghdx.healthdata.org/gbd-2019/data-input-sources [14].

### Data processing and disease model

Where possible, the prevalence estimates were divided by age and sex. If studies reported the prevalence for wide age groups by sex (e.g., among 20 to 55 year old males and 20 to 55 year old females), or by specific age groups with the two sexes combined (e.g., prevalence in 15 to 30 year olds, for males and females together), age-specific estimates were split by sex using the sex ratio reported and the bounds of uncertainty. However, if the prevalence data for both sexes could not be split using a within-study sex ratio, they were split using a sex ratio produced from a meta-analysis of existing sex-specific data using Meta-Regression with Bayesian Priors, Regularisation, and Trimming (MR-BRT). There was a ratio of females to males of 1.90 (1.85 to 1.96). Lastly, bias adjustments were made for studies which reported estimates across large age groups (i.e., 25 years or more). These were partitioned into five-year age groups using the prevalence age pattern estimated by the most accurate Bayesian meta-regression models (DisMod-MR 2.1) that were found in GBD 2017, for each headache type. The prevalence or incidence of TTH was estimated using DisMod-MR 2.1.

The DisMod settings for TTH included the assumptions of no excess mortality and no cases prior to 5 years old. Separate DisMod models were produced for definite TTH, probable TTH, and the total TTH category, with an upper remission bound of 0.5 for all three. For consistency, the modelled results for the probable and definite TTH were then scaled to fit the total TTH envelope. Several data sources only reported definite TTH, particularly those published before the ICHD became the standard (the criteria were established in 1988). Those studies that only reported definite TTH were also adjusted to the total TTH category, in order to better inform the model. Following this, those studies that reported both definite and total TTH were added to the regression models, by sex, in order to produce age- and sex-specific adjustments. The sex-specific models produced an implausible age pattern for females, which was the reverse of the original data. Therefore, a regression model was run to derive an age-specific adjustment that was used for both males and females [14].

In GBD 2019, data from the meta-analysis Lifting the Burden was used to provide the symptomatic time for TTH, as well as estimates for probable, definite, and total TTH. Using the Meta-Regression with Bayesian Priors, Regularisation, and Trimming (MR-BRT), the proportion of symptomatic time was calculated to be 0.029 for definite TTH and 0.0.021 for probable TTH [14].

### Years lived with disability

The GBD disability weight survey provides lay descriptions of sequelae which highlight major functional consequences and symptoms [15]. Probable and definite TTH was defined as “has a moderate headache that also affects the neck, which causes difficulty in daily activities.” and had a disability weight of 0.037 (95% confidence interval: 0.022 to 0.057), which means that a person experiences a 3.7% health loss during an attack, compared to a person in full health [15]. However, there were no lay descriptions or disability weights for asymptomatic TTH.

The DALY is a standard metric used to quantify the burden of a disease or disorder [16], and is produced by combining the years of life lost due to premature mortality and the YLDs. As there was no evidence that TTH caused any mortality, the YLD and DALY estimates were assumed to be the same [16]. The YLDs for TTH were calculated using the point prevalence and *time symptomatic*, multiplied by the appropriate disability weight. Uncertainty intervals (95%) were also produced by sampling 1,000 draws at every computational step, as well as by combining uncertainty from multiple sources (e.g., input data, corrections of measurement error, and estimates of residual non-sampling error). The uncertainty intervals (UIs) were comprised of the 2.5 and 97.5 percentile values of the ordered draws.

### Compilation of the results

Smoothing splines were used to investigate the relationship between the TTH burden, as measured by YLDs, and SDI [17]. The SDI is comprised of: i) the lag-distributed income per capita, which is the gross domestic product per capita smoothed over the preceding decade; ii) average number of years of schooling for the population aged above 15 years old; and iii) the total fertility rate for those under 25 years old. The index ranges from 0-1, which denote less developed to most developed, respectively. The age-standardised point prevalence, annual incidence and YLD rates were presented using R software, version 3.5.2.

## Results

### The Middle East and North Africa region

In 2019, there were an estimated 149.1 million (95% UI: 128.5 to 171.0) prevalent cases of TTH in the MENA region, with an age-standardised point prevalence of 24504.5 (21304.8 to 27987.5) per 100000 population, which represents a 2.0% increase since 1990 (0.7 to 3.4) (Table 1 and Table S1). TTH accounted for 52.9 million (46.1 to 59.5) incident cases in 2019, with an age-standardised rate of 8680.1 (7631.6 to 9732.5) per 100000 population, which is 0.9% higher than in 1990 (-0.1 to 1.7) (Table 1 and Table S2). In 2019, the number of regional YLDs was 416.6 thousand (138.3 to 1196.8), with an age-standardised rate of 68.1 (22.8 to 195.5) YLDs per 100000 population, an increase of 1.0% since 1990 (-9.5 to 8.7) (Table 1 and Table S3).

**Table 1:** Prevalent cases, incident cases and YLDs due to tension-type headache in 2019 and percentage change of age-standardised rates during 1990-2019

	Prevalence (95% UI)			Incidence (95% UI)			YLDs (95% UI)		
	Counts (2019)	ASRs (2019)	Pcs in ASRs 1990-2019	Counts (2019)	ASRs (2019)	Pcs in ASRs 1990-2019	Counts (2019)	ASRs (2019)	Pcs in ASRs 1990-2019
**North Africa and Middle East**	**149061721 (128455947 , 170990926)**	**24504.5 (21304.8 , 27987.5)**	**2 (0.7 , 3.4)**	**52879780 (46137009 , 59485212)**	**8680.1 (7631.6 , 9732.5)**	**0.9 (-0.1 , 1.7)**	**416595 (138258 , 1196825)**	**68.1 (22.8 , 195.5)**	**1 (-9.5 , 8.7)**
**Afghanistan**	**7750880 (6436495 , 9231384)**	**23693.8 (20386.8 , 27306.9)**	**0.4 (0.1 , 0.7)**	**2938263 (2512679 , 3388697)**	**8515.2 (7457.9 , 9593.9)**	**0.4 (0.2 , 0.6)**	**19586 (6272 , 57731)**	**65.2 (22.1 , 183.3)**	**-0.1 (-9.6 , 7.9)**
**Algeria**	**9888031 (8443602 , 11527577)**	**23647.6 (20341.2 , 27276.9)**	**-0.1 (-0.1 , 0)**	**3544462 (3089897 , 4021845)**	**8501 (7440.7 , 9578.6)**	**-0.1 (-0.1 , -0.1)**	**28460 (9650 , 79524)**	**66.7 (22.6 , 189)**	**0.3 (-10.2 , 7.9)**
**Bahrain**	**377845 (318458 , 442847)**	**23716 (20422 , 27274.1)**	**-0.1 (-0.3 , 0.1)**	**130833 (112605 , 149916)**	**8524.4 (7476 , 9592.8)**	**-0.2 (-0.3 , 0)**	**1117 (386 , 3084)**	**64.6 (21.3 , 189.3)**	**-0.3 (-10.7 , 7.7)**
**Egypt**	**25094802 (21538924 , 28759918)**	**26290.9 (22878.1 , 29775.3)**	**3.6 (-1.6 , 9.5)**	**8593585 (7460381 , 9681319)**	**8812.2 (7762.7 , 9893.9)**	**0.7 (-2.7 , 5)**	**65173 (20989 , 196744)**	**69.1 (22.6 , 207.8)**	**1.4 (-10.1 , 10.8)**
**Iran (Islamic Republic of)**	**25772952 (22795090 , 28770049)**	**29640.4 (26202.1 , 32949.4)**	**9.7 (6.5 , 13.1)**	**8430126 (7411247 , 9501060)**	**9837.5 (8687.5 , 11030.7)**	**5.3 (1.9 , 7.9)**	**70182 (23015 , 207136)**	**77.6 (24.9 , 236.4)**	**5.2 (-5.7 , 15.9)**
**Iraq**	**9672804 (8160231 , 11338809)**	**23667.9 (20363.1 , 27281.6)**	**0 (0 , 0)**	**3527971 (3048007 , 4011221)**	**8507.1 (7448.5 , 9584.3)**	**0 (0 , 0)**	**26343 (8663 , 77009)**	**66.2 (22.1 , 190.3)**	**0.7 (-11.1 , 9.1)**
**Jordan**	**2700147 (2285860 , 3158502)**	**23661.5 (20365.4 , 27259.2)**	**0.1 (0 , 0.2)**	**982482 (849563 , 1113702)**	**8508.4 (7455 , 9588.3)**	**0.1 (0 , 0.2)**	**7406 (2514 , 21769)**	**66.2 (22.7 , 191.4)**	**0.1 (-10.9 , 8.3)**
**Kuwait**	**1117900 (946810 , 1313413)**	**23533 (20134.8 , 26965.1)**	**-0.9 (-4.9 , 3)**	**391309 (340667 , 446614)**	**8487.2 (7489.2 , 9540.2)**	**-0.6 (-3.3 , 2.4)**	**3205 (1084 , 8967)**	**62.8 (20.8 , 186.4)**	**1.5 (-10.4 , 11.9)**
**Lebanon**	**1237281 (1062030 , 1432419)**	**23655 (20353.1 , 27281.3)**	**0.1 (0 , 0.3)**	**441957 (387091 , 499441)**	**8503.8 (7444.7 , 9581.2)**	**0.2 (0.1 , 0.3)**	**3565 (1217 , 10006)**	**67 (22.7 , 191.7)**	**0.8 (-10.8 , 9.4)**
**Libya**	**1698733 (1444446 , 1978942)**	**23667.9 (20357.9 , 27281.4)**	**-0.1 (-0.2 , 0.1)**	**599202 (520188 , 678679)**	**8508.4 (7450.6 , 9585.8)**	**-0.1 (-0.2 , 0.1)**	**4920 (1694 , 13899)**	**66.4 (22.8 , 191)**	**1.1 (-11.2 , 9.5)**
**Morocco**	**8691626 (7444134 , 10049060)**	**23654.8 (20352.1 , 27282.8)**	**0 (-0.1 , 0)**	**3107802 (2711391 , 3502989)**	**8502.5 (7441.7 , 9579.2)**	**0 (0 , 0)**	**24818 (8463 , 69426)**	**66.5 (22.7 , 187.3)**	**0.2 (-9.8 , 8.1)**
**Oman**	**1138730 (948472 , 1361335)**	**23692.5 (20423.5 , 27194.2)**	**-0.1 (-0.3 , 0)**	**403518 (342668 , 467602)**	**8531.4 (7483.3 , 9610.9)**	**0 (-0.2 , 0.1)**	**3196 (1042 , 8985)**	**64.3 (21.6 , 190.1)**	**0 (-15.4 , 10.8)**
**Palestine**	**1094411 (920963 , 1287920)**	**23661.8 (20360.3 , 27277.7)**	**0 (-0.2 , 0.1)**	**405495 (349922 , 462326)**	**8503.8 (7444.3 , 9580.2)**	**-0.1 (-0.2 , 0)**	**2914 (952 , 8613)**	**66.2 (22.1 , 187.5)**	**-0.3 (-13 , 9.3)**
**Qatar**	**756418 (622260 , 910774)**	**23538.3 (20220.1 , 27033.6)**	**-0.5 (-0.9 , -0.2)**	**263229 (222394 , 306753)**	**8485.2 (7444.1 , 9569.2)**	**-0.4 (-0.7 , -0.1)**	**2135 (699 , 6004)**	**62 (20.7 , 184.7)**	**-1.4 (-11.6 , 7.2)**
**Saudi Arabia**	**8354742 (7033634 , 9767868)**	**21757.9 (18684.4 , 24972.1)**	**-2.7 (-7 , 2)**	**3081911 (2653414 , 3512212)**	**8206.1 (7168.6 , 9215.6)**	**-1 (-4 , 2)**	**25584 (9050 , 70083)**	**63 (22 , 180.3)**	**-0.3 (-18.6 , 10.8)**
**Sudan**	**8893989 (7481341 , 10475764)**	**23598.8 (20291 , 27242.1)**	**-0.1 (-0.2 , -0.1)**	**3301315 (2843973 , 3772470)**	**8482.4 (7418.8 , 9558.5)**	**-0.1 (-0.2 , -0.1)**	**23633 (7813 , 69143)**	**66.4 (23.2 , 189)**	**0.5 (-11.8 , 9.8)**
**Syrian Arab Republic**	**3507816 (2996722 , 4066438)**	**23640.6 (20306.7 , 27263.7)**	**0 (-0.3 , 0.3)**	**1259310 (1101071 , 1426066)**	**8488 (7422.9 , 9561.3)**	**-0.1 (-0.3 , 0.1)**	**9810 (3361 , 28381)**	**67 (22.9 , 189.7)**	**1.1 (-10.5 , 9.5)**
**Tunisia**	**2863725 (2464872 , 3296804)**	**23657.2 (20350.7 , 27289)**	**0.1 (0 , 0.1)**	**1013213 (890081 , 1140492)**	**8502 (7441.4 , 9578.4)**	**0 (0 , 0.1)**	**8414 (2880 , 23528)**	**67 (22.6 , 192.5)**	**0.7 (-10.7 , 8.5)**
**Turkey**	**19035787 (16409964 , 21823161)**	**21726.3 (18745.7 , 24838.8)**	**1 (-2.9 , 5.2)**	**7018674 (6183837 , 7932907)**	**8197.3 (7199.6 , 9252.4)**	**0.7 (-2.5 , 3.6)**	**60666 (21747 , 160117)**	**66.6 (23.6 , 180)**	**0.8 (-10 , 10.1)**
**United Arab Emirates**	**2512643 (2048289 , 3008501)**	**23676 (20386.9 , 27204.5)**	**-0.2 (-0.5 , 0)**	**863143 (722148 , 1015406)**	**8528.1 (7490.2 , 9612.7)**	**-0.1 (-0.3 , 0.1)**	**7426 (2396 , 20517)**	**63.2 (20.7 , 187.8)**	**0 (-12.6 , 8.9)**
**Yemen**	**6749015 (5678757 , 8023953)**	**23663 (20359.2 , 27287.1)**	**0 (-0.1 , 0.1)**	**2528256 (2163210 , 2899036)**	**8504.5 (7443.4 , 9580.9)**	**0 (0 , 0.1)**	**17617 (5766 , 51725)**	**66.1 (22.8 , 185.6)**	**0.6 (-11.1 , 9.8)**

### National level

**Fig. 1:**
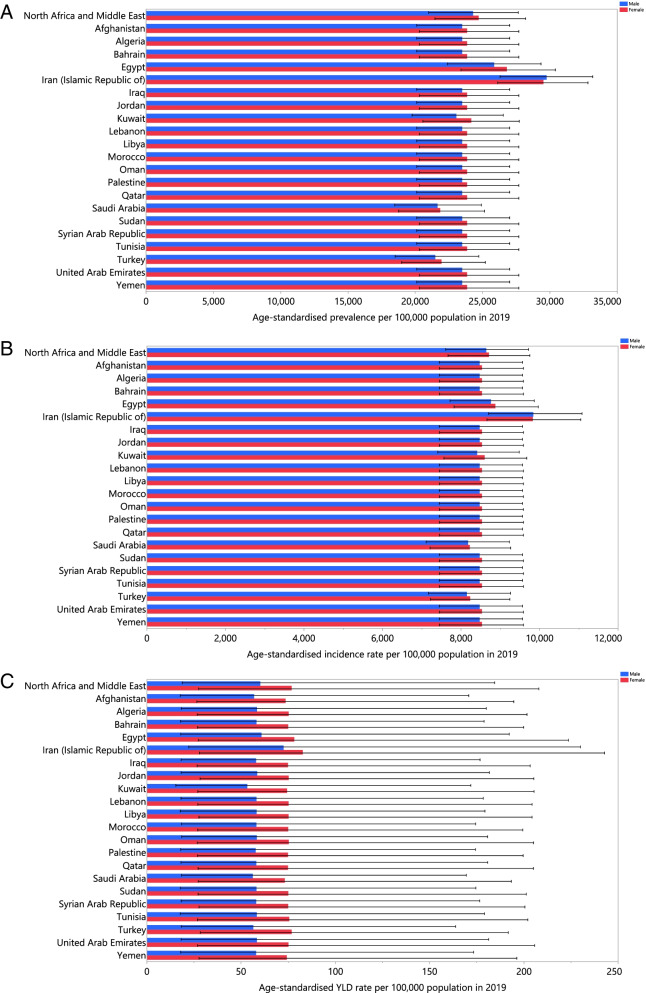
Age-standardised point prevalence (**A**), incidence (**B**), and YLDs (**C**) of tension-type headache (per 100000 population) in the Middle East and North Africa region in 2019, by sex and country. YLD= years lived with disability. (Generated from data available from http://ghdx.healthdata.org/gbd-results-tool).

In 2019, the national age-standardised point prevalence of TTH ranged from 21726.3 to 29640.4 cases per 100000 population, among the countries that comprise the MENA region. Iran [29640.4 (26202.1 to 32949.4)], Egypt [26290.9 (22878.1 to 29775.3)] and Bahrain [23716.0 (20422.0 to 27274.1)] had the three highest age-standardised point prevalences of TTH in 2019. In contrast, Turkey [21726.3 (18745.7 to 24838.8)], Saudi Arabia [21757 (18684.4 to 24972.1)] and Kuwait [23533.0 (20134.8 to 26965.1)] had the lowest (Fig. 1A and Table S1).

The national age-standardised incidence rates of TTH in 2019 ranged from 8197.3 to 9837.5 cases per 100000 population. The highest rates were observed in Iran [9837.5 (8687.5 to 11030.7)], Egypt [8812.2 (95% UI: 7762.7 to 9893.9)] and Oman [8531.4 (7483.3 to 9610.9)], while the lowest were found in Turkey [8197.3 (7199.6 to 9252.4)], Saudi Arabia [8206.1 (7168.6 to 9215.6)] and Sudan [8482.4 (7418.8 to 9558.5)] (Fig. 1B and Table S2).

In 2019, the national age-standardised YLD rate of TTH ranged from 62.0 to 77.6 cases per 100000 population among the MENA countries. The highest rates were observed in Iran [77.6 (24.9 to 236.4)], Egypt [69.1 (22.6 to 207.8)] and Tunisia [67.0 (22.6 to 192.5)]. Conversely, the lowest rates were seen in Qatar [62.0 (20.7 to 184.7)], Kuwait [62.8 (20.8 to 186.4)] and Saudi Arabia [63.0 (22.0 to 180.3)] (Fig. 1C and Table S3).

The percentage change in the age-standardised point prevalence, from 1990 to 2019, did not change significantly in most MENA countries. Nevertheless, Iran [9.7% (6.5 to 13.1)] and Saudi Arabia [-2.7% (-7.0 to 2.0)] had the highest increases and decreases in the age-standardised point prevalence, respectively (Table S1 and Fig. S1).

In addition, there were no substantial changes in the age-standardised incidence rates for most countries in MENA, except for Iran which had the highest change [5.3% (1.9 to 7.9)] (Table S2 and Fig. S2). There was also a similar pattern for the age-standardised YLD rate among the MENA countries (Table S3 and Fig. S3).

### Age and sex patterns

**Fig. 2:**
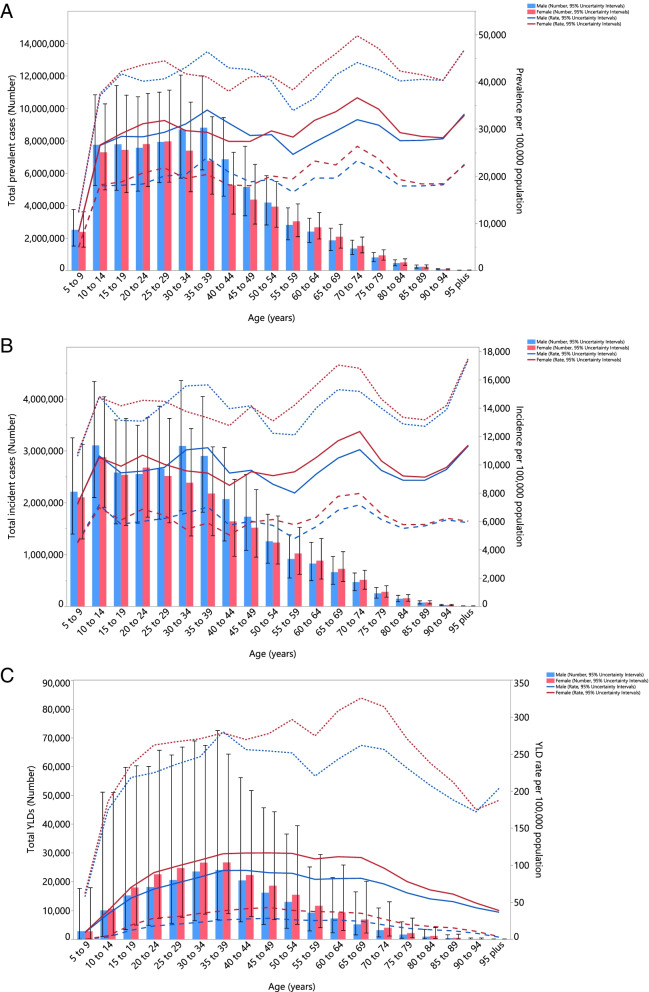
Numbers of prevalent cases and prevalence (**A**), number of incident cases and incidence rate (**B**) and the number of YLDs and YLD rate (**C**) for tension-type headache per 100000 population in the Middle East and North Africa region, by age and sex in 2019; Dotted and dashed lines indicate 95% upper and lower uncertainty intervals, respectively. YLD= years lived with disability. (Generated from data available from http://ghdx.healthdata.org/gbd-results-tool).

In 2019, the regional point prevalence of TTH was highest in the 35-39 and 70-74 age groups, for males and females, respectively. Also, the number of prevalent cases was estimated to be highest in those aged 35-39 and 25-29 years, in both males and females, respectively. However, there was no discernible difference in the prevalence of TTH between males and females (Fig. 2A).

In 2019, the number of incidences reached its highest in the 10-14 age group, while the age-standardised incidence rates were highest in the 95^+^ and 70-74 age groups in males and females, respectively (Fig. 2B).

There was an increase in the regional YLD rate of TTH for females up to the 65-69 age group, followed by a decrease. In contrast, the regional YLD rate for males increased with advancing age up to the 35-39 age group, then it decreased, except for an increase in those aged 55-69 years old. In addition, the number of YLDs was highest in the 35-39 age group, for both males and females (Fig. 2C).

**Fig 3:**
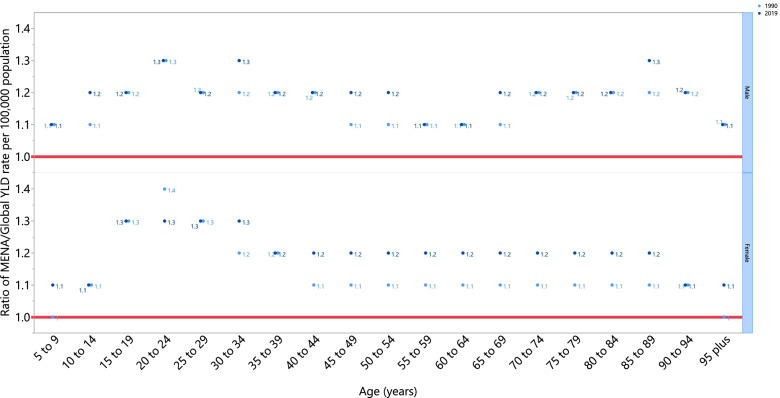
Ratio of the Middle East and North Africa region to the global tension-type headache YLD rate according to age group and sex, 1990–2019. YLD= years lived with disability. (Generated from data available from http://ghdx.healthdata.org/gbd-results-tool).

The rate ratio of TTH, which compares the age-standardised YLD rates in MENA to the global rates, by sex and for each age group, showed that MENA had higher age-standardised rates in 1990 and 2019 across all age groups and in both sexes. In 1990, the ratio was highest in the 20-24 age group, in both males (1.3) and females (1.4). Moreover, also in 2019, males aged 20-24, 30-34, and 85-89 years old had the highest ratio (1.3), while for females the ratio was highest in the 15-34 age group (1.3) (Fig. 3).

### Association with the Socio-demographic Index (SDI)

**Fig. 4:**
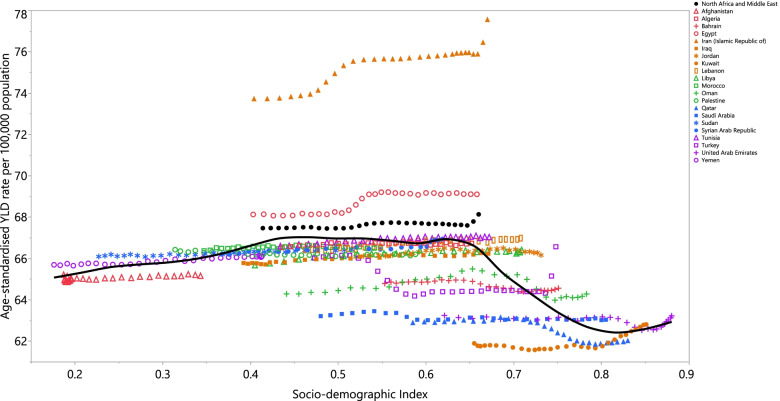
Age-standardised YLD rates of tension-type headache for 21 countries and territories, by SDI in 2019; Expected values based on the Socio-demographic Index and disease rates in all locations are shown as the black line. Each point shows the observed age-standardised YLD rate for each country in 2019. YLD= years lived with disability. SDI= Socio-demographic Index (Generated from data available from http://ghdx.healthdata.org/gbd-results-tool).

The burden of TTH was not observed to have a clear association with SDI. However, a slight rising trend in the burden of TTH was observed with increasing development status up to the SDI level of 0.65. Then, it sharply decreased reaching to the lowest YLD rate in the SDI level of 0.8, following by an increase for the remaining SDI levels. Countries such as Iran and Egypt had higher than expected burdens, whereas countries like the Syrian Arab Republic, Qatar and Afghanistan had lower than expected burdens (Fig. 4).

## Discussion

The present study is the most comprehensive analysis of the burden attributable to TTH in the MENA region. The age-standardised point prevalence per 100000 population was 24504.5 in 2019, which has increased by 2.0% since 1990. Moreover, TTH accounted for 8680.1 and 68.1 age-standardised incidence and YLD rates, respectively, which did not change greatly from 1990 to 2019. It is important to note that we found the burden attributable to TTH in the MENA region was larger than the corresponding global burden for both sexes and in all age groups. Therefore, investigating and understanding the level of disability caused by TTH might help policy makers, funding organisations, and the pharmaceutical industry in developing successful strategies for mitigating the burden of TTH and to allow an efficient allocation of resources.

Globally, TTH is the most prevalent neurological disorder and the third most prevalent disorder. According to reports from the GBD 2016 and 2017 studies, the age-standardised point prevalence of TTH has decreased since 1990 [7, 8, 18]. At regional level, the GBD 2016 study reported an 8.5% reduction in the age-standardised point prevalence of TTH in the MENA region since 1990 [7]. In line with this finding, research in the Eastern Mediterranean Region (EMR) observed a similar decrease in the point prevalence of TTH from 1990 to 2016 [19]. These findings are in contrast to our research, which found a 2.0% increase. These differences may be due to variations in the methodologies used by the GBD 2016 and GBD 2019 projects.

Although no change was observed in the age-standardised YLD rates, the number of YLDs increased between 1990 and 2019. This potentially reflects population growth and changes in the age distribution, which has shifted towards a lower number of children and adolescents, as well as more young and middle aged adults. The recently stable YLD rates could indicate that no progress has been made in the treatment options and therapeutic approaches for increasing the quality of life for patients suffering from TTH. Additionally, the constant burden of TTH may be due to unchanged risk factors or underlying causes. Furthermore, access to health care facilities, and in particular the availability of analgesic medications as the most common headache-abortive agents for TTH, could further affect the rates of disability, which have not improved in the region since 1990, especially for low-income countries. A recent publication evaluated the factors affecting the health related quality of life (HRQoL) in TTH patients. They found that the incidence of TTH decreased the HRQoL of the affected patients. Furthermore, among TTH patients, age, female sex, and poor self-rated health were all associated with lower physical HRQoL, but depression was the only factor associated with lower mental HRQoL [20].

The age-standardised YLD rates due to TTH were higher in the MENA region than the corresponding global rates for both sexes and across all age groups. It has been postulated that anxiety and depression, as substantial and complicating comorbidities of TTH, play a key role in the exacerbation of headache symptoms and thus cause further significant disabilities [1, 20, 21]. A potential justification for the greater burden of TTH in the MENA region could be due to the higher incidence of several psychiatric comorbidities. According to recent GBD studies, the age-standardised incidence rate of depression and anxiety in MENA is around 30% higher than the global average [22, 23]. The present research found that Iran had the largest burden of TTH in MENA. Interestingly, previous research found Iran had the highest age-standardised incidence rates for both anxiety and depression [22, 23]. There may be a number of reasons for this, including the eight-year-war with Iraq, a poorly performing economy and international sanctions that targeted all sectors of Iran’s economy over the past three decades, resulting in a surge in the burden of psychological stress among the Iranian population [24, 25]. These findings highlight the urgent need for policy interventions which target these psychological comorbidities more effectively, in order to help reduce the disabilities caused by TTH.

Our findings showed that the TTH attributable YLD rate was higher for females than for males. Similar findings were obtained from worldwide data in 2016, where females had a 50% higher YLD rate than males. Furthermore, TTH was found to cause 0.9% of all YLDs across the globe, while the corresponding values were 1.0% for females and 0.8% for males [7]. Moreover, in a study aiming to measure the global burden of TTH in children and adolescent, females were responsible for the higher proportion of attributable burden [26]. Another recent study came to the conclusion that psychological comorbidities, such as depression, were the cause of the larger burden of TTH among females [27]. Moreover, another study concluded that female TTH sufferers tended to experience more intensive pain, depressive symptoms and a lower quality of life [28]. Consequently, more integrative planning to control the comorbidities of TTH in females might be helpful in addressing the corresponding burden.

We found that the number of prevalent, incidence and YLD cases attributable to TTH reached its peak in younger and middle-aged adults, and this declined with age. In accordance with our findings, globally TTH has been found to be particularly burdensome in those aged 15-49 years old [7, 18]. Although the majority of non-communicable diseases cause more disabilities in older ages, the finding that TTH affects younger people suggests that TTH may cause losses in productive work time, therefore leading to a huge economic burden which affects not only the individuals, but society as a whole [29–31]. In line with this, a study from Denmark found that in the previous year 12% of those employed had suffered from TTH and had missed at least one day of work because of a headache. Therefore, the total number of workdays lost due to TTH was estimated to be 820 days per 1000 employees per year [32].

The SDI level of the countries in the MENA region was not a major contributor to the size of the TTH burden, during the period 1990-2019. A similar trend was observed at the global level and also for the EMR countries [19, 33]. However, we found that the burden of TTH was slightly increased up to the SDI level of 0.65 and then decreased sharply for the remaining SDI levels. This finding might imply that the demographic, epidemiological and health transitions currently facing the world, and particularly in MENA [16], did not substantially affect the burden of TTH in low- and middle-income countries over the past three decades, while remarkably reduced the burden of TTH in high-income countries. Thus, it could be anticipated that the relative importance of TTH will increase in the near future for low- and middle-income countries, as the YLD rates for several disorders (e.g., infections, nutritional deficiencies, maternal and neonatal diseases and many non-communicable disorders) are greatly decreasing among these countries.

TTH is the most prevalent type of headache and has become a major public health concern, due to the disabilities imposed upon the population. However, the realisation that headache disorders are debilitating for a large number of affected people has been hindered by the facts that TTH does not caused death or a permanent and obvious disability, and many people routinely experience headaches [34, 35]. Furthermore, research has shown that subjects with TTH (16%) attend medical centers much less frequently than those with migraine (56%) [32]. Therefore, more attention should focus on improving the awareness of patients and providing education for healthcare personnel regarding simple TTH remedies and practices for the treatment of acute attacks and the prevention of further episodes. Moreover, since there is no specific and effective cure for TTH, it is necessary to allocate more resources to understanding the pathophysiology of this type of headache and thus discovering innovative treatments and preventive strategies. Concurrently, healthcare services should do a better job at reaching people with cost-effective medications to relieve pain and reduce the disabilities due to TTH, improving productivity at work for those effected.

We acknowledge present study has some limitations that must be considered when interpreting our findings. Firstly, restrictions in data sources is still a potential shortcoming of the headache burden estimates [7]. Furthermore, data about headaches is not accurately collected in many national health surveys. Therefore, the estimation of TTH would benefit from more accurate data gathering and registration in many health systems, preferably with definite time intervals. Secondly, several risk factors have been linked to the emergence of headaches, such as stress, anxiety, depression and sleep apnea, although the evidence for their impact is inadequate [20, 36–38]. It is interesting to note that these risk factors have not yet been included as risk factors in the GBD study [33] and should be evaluated against GBD criteria, as to whether they provide convincing or probable evidence of causation. Thirdly, the burden estimates of TTH may vary greatly across different countries in the MENA region, owing in part to differences in race, ethnicity and other demographic features of these populations [39]. Fourthly, we were not able to stratify our results according to the type of TTH, which are episodic TTH (ETTH) and chronic TTH (CTTH). However, the burden of headache has been found to be higher in CTTH than for ETTH patients [40]. The reliability and validity of our findings may be affected by several factors that necessitate high methodological quality investigations to be conducted in the future. The next round of GBD studies should incorporate new data sources from additional countries and with less methodological heterogeneity, which would help to obtain stronger evidence and the ability to draw more realistic conclusions.

## Conclusion

The present study has shown that TTH causes a large burden in the MENA region, in terms of disabilities and loss of health, and that the burden in MENA is higher than the global level for both sexes and all age groups. Therefore, prevention of TTH could help alleviate the attributable burden imposed on the hundreds of millions of people suffering from TTH around the region. Future high quality epidemiological studies at the national and subnational levels, which are calibrated to the local settings in MENA, are needed to strengthen the certainty and validity of the estimated burden.

## Supplementary Information

**Additional file 1: Table S1.** Prevalence of tension-type headache in 1990 and 2019 for both sexes and the percentage change in the age-standardised rates (ASRs) per 100000 in the North Africa and the Middle East region (Generated from data available from http://ghdx.healthdata.org/gbd-results-tool). **Table S2.** Incidence of tension-type headache in 1990 and 2019 for both sexes and the percentage change in the age-standardised rates (ASRs) per 100000 in the Middle East and North Africa region (Generated from data available from http://ghdx.healthdata.org/gbd-results-tool). **Table S3.** YLDs due to tension-type headache in 1990 and 2019 for both sexes and the percentage change in the age-standardised rates (ASRs) per 100000 in the Middle East and North Africa region (Generated from data available from http://ghdx.healthdata.org/gbd-results-tool). **Figure S1.** The percentage change in the age-standardised point prevalence of tension-type headache in the Middle East and North Africa region from 1990 to 2019, by sex and country. (Generated from data available from http://ghdx.healthdata.org/gbd-results-tool). **Figure S2.** The percentage change in the age-standardised incidence of tension-type headache in the Middle East and North Africa region from 1990 to 2019, by sex and country. (Generated from data available from http://ghdx.healthdata.org/gbd-results-tool). **Figure S3.** The percentage change in the age-standardised YLDs of tension-type headache in the Middle East and North Africa region from 1990 to 2019, by sex and country. YLD= years lived with disability. (Generated from data available from http://ghdx.healthdata.org/gbd-results-tool).

## Data Availability

The data used for these analyses are all publicly available at http://ghdx.healthdata.org/gbd-results-tool.
