# Correction: Burden of tension-type headache in the Middle East and North Africa region, 1990-2019

**DOI:** 10.1186/s10194-022-01454-4

**Published:** 2022-08-02

**Authors:** Saeid Safiri, Ali-Asghar Kolahi, Maryam Noori, Seyed Aria Nejadghaderi, Armin Aslani, Mark J. M. Sullman, Mehdi Farhoudi, Mostafa Araj-Khodaei, Gary S. Collins, Jay S. Kaufman, Kurosh Gharagozli

**Affiliations:** 1 Neurosciences Research Center, Aging Research Institute, Tabriz University of Medical Sciences, Tabriz, Iran; 2 Social Determinants of Health Research Center, Department of Community Medicine, Faculty of Medicine, Tabriz University of Medical Sciences, Tabriz, Iran; 3 Social Determinants of Health Research Center, Shahid Beheshti University of Medical Sciences, Tehran, Iran; 4 Student Research Committee, School of Medicine, Iran University of Medical Sciences, Tehran, Iran; 5 Urology Research Center, Tehran University of Medical Sciences, Tehran, Iran; 6 Research Center for Integrative Medicine in Aging, Aging Research Institute, Tabriz University of Medical Sciences, Tabriz, Iran; 7 Systematic Review and Meta-analysis Expert Group (SRMEG), Universal Scientific Education and Research Network (USERN), Tehran, Iran; 8 Department of Life and Health Sciences, University of Nicosia, Nicosia, Cyprus; 9 Department of Social Sciences, University of Nicosia, Nicosia, Cyprus; 10 Centre for Statistics in Medicine, NDORMS, Botnar Research Centre, University of Oxford, Oxford, UK; 11 NIHR Oxford Biomedical Research Centre, Oxford University Hospitals NHS Foundation Trust, Oxford, UK; 12 Department of Epidemiology, Biostatistics and Occupational Health, Faculty of Medicine, McGill University, Montreal, QC Canada; 13 Brain Mapping Research Center, Shahid Beheshti University of Medical Sciences, Tehran, Iran

**The Journal of Headache and Pain 23, 77 (2022)**

**https://doi.org/10.1186/s10194-022-01445-5**

After publication of this article [1], the authors reported that in the Methods section, several citations to references were incorrect. the citation to [8, 12, 13] should have read [12-14], and the citations to [14] should have read [12].

The original article [1] has been corrected.
