# Supplementary material for: Burden of tension-type headache in the Middle East and North Africa region, 1990-2019
Source: J Headache Pain. 2022 Jul 6;23(1):77. doi: 10.1186/s10194-022-01445-5 (PMC9258079; doi:10.1186/s10194-022-01445-5)
Supplement: Supplementary file 1 — Additional file 1: Table S1. Prevalence of tension-type headache in 1990 and 2019 for both sexes and the percentage change in the age-standardised rates (ASRs) per 100000 in the North Africa and the Middle East region (Generated from data available from http://ghdx.healthdata.org/gbd-results-tool). Table S2. Incidence of tension-type headache in 1990 and 2019 for both sexes and the percentage change in the age-standardised rates (ASRs) per 100000 in the Middle East and North Africa region (Generated from data available from http://ghdx.healthdata.org/gbd-results-tool). Table S3. YLDs due to tension-type headache in 1990 and 2019 for both sexes and the percentage change in the age-standardised rates (ASRs) per 100000 in the Middle East and North Africa region (Generated from data available from http://ghdx.healthdata.org/gbd-results-tool). Figure S1. The percentage change in the age-standardised point prevalence of tension-type headache in the Middle East and North Africa region from 1990 to 2019, by sex and country. (Generated from data available from http://ghdx.healthdata.org/gbd-results-tool). Figure S2. The percentage change in the age-standardised incidence of tension-type headache in the Middle East and North Africa region from 1990 to 2019, by sex and country. (Generated from data available from http://ghdx.healthdata.org/gbd-results-tool). Figure S3. The percentage change in the age-standardised YLDs of tension-type headache in the Middle East and North Africa region from 1990 to 2019, by sex and country. YLD= years lived with disability. (Generated from data available from http://ghdx.healthdata.org/gbd-results-tool). [file 10194_2022_1445_MOESM1_ESM.zip › Supplementary Table 1, Prevalence, TTH, MENA.docx]

| **Table S1: Prevalence of tension-type headache in 1990 and 2019 for both sexes and percentage change in age-standardised rates (ASRs) per 100,000 in the North Africa and the Middle East region**  **(Generated from data available from http://ghdx.healthdata.org/gbd-results-tool)** | | | | | |
| --- | --- | --- | --- | --- | --- |
|  | **1990** | | **2019** | | **Percentage change in ASRs per 100,000** |
|  | **No (95% UI)** | **ASRs per 100,000 (95% UI)** | **No (95% UI)** | **ASRs per 100,000 (95% UI)** |  |
| **North Africa and Middle East** | **73709140 (62670234 , 85870898)** | **24019.1 (20753.8 , 27386.5)** | **149061721 (128455947 , 170990926)** | **24504.5 (21304.8 , 27987.5)** | **2 (0.7 , 3.4)** |
| **Afghanistan** | **2316475 (1950146 , 2722335)** | **23599.5 (20275 , 27230.6)** | **7750880 (6436495 , 9231384)** | **23693.8 (20386.8 , 27306.9)** | **0.4 (0.1 , 0.7)** |
| **Algeria** | **5330721 (4470318 , 6296212)** | **23667.7 (20359.4 , 27292.4)** | **9888031 (8443602 , 11527577)** | **23647.6 (20341.2 , 27276.9)** | **-0.1 (-0.1 , 0)** |
| **Bahrain** | **116519 (97672 , 137988)** | **23734.6 (20456.3 , 27270.3)** | **377845 (318458 , 442847)** | **23716 (20422 , 27274.1)** | **-0.1 (-0.3 , 0.1)** |
| **Egypt** | **12731883 (10691991 , 14902978)** | **25385.8 (21788 , 29204.6)** | **25094802 (21538924 , 28759918)** | **26290.9 (22878.1 , 29775.3)** | **3.6 (-1.6 , 9.5)** |
| **Iran (Islamic Republic of)** | **13686833 (11848934 , 15677599)** | **27017.1 (23937.5 , 30258.3)** | **25772952 (22795090 , 28770049)** | **29640.4 (26202.1 , 32949.4)** | **9.7 (6.5 , 13.1)** |
| **Iraq** | **3531873 (2959383 , 4180434)** | **23673.5 (20365.6 , 27287.3)** | **9672804 (8160231 , 11338809)** | **23667.9 (20363.1 , 27281.6)** | **0 (0 , 0)** |
| **Jordan** | **775192 (643880 , 925960)** | **23643.1 (20343.6 , 27253.9)** | **2700147 (2285860 , 3158502)** | **23661.5 (20365.4 , 27259.2)** | **0.1 (0 , 0.2)** |
| **Kuwait** | **406259 (340704 , 482157)** | **23749.8 (20377.9 , 27300)** | **1117900 (946810 , 1313413)** | **23533 (20134.8 , 26965.1)** | **-0.9 (-4.9 , 3)** |
| **Lebanon** | **704747 (598774 , 821082)** | **23624.9 (20315.7 , 27263.8)** | **1237281 (1062030 , 1432419)** | **23655 (20353.1 , 27281.3)** | **0.1 (0 , 0.3)** |
| **Libya** | **871464 (730034 , 1031393)** | **23682.3 (20386.7 , 27272)** | **1698733 (1444446 , 1978942)** | **23667.9 (20357.9 , 27281.4)** | **-0.1 (-0.2 , 0.1)** |
| **Morocco** | **5492606 (4631617 , 6449427)** | **23656.2 (20348.8 , 27282.1)** | **8691626 (7444134 , 10049060)** | **23654.8 (20352.1 , 27282.8)** | **0 (-0.1 , 0)** |
| **Oman** | **405014 (339039 , 479703)** | **23721.4 (20441.2 , 27238.4)** | **1138730 (948472 , 1361335)** | **23692.5 (20423.5 , 27194.2)** | **-0.1 (-0.3 , 0)** |
| **Palestine** | **401906 (333029 , 477777)** | **23668.9 (20371.1 , 27289.2)** | **1094411 (920963 , 1287920)** | **23661.8 (20360.3 , 27277.7)** | **0 (-0.2 , 0.1)** |
| **Qatar** | **105850 (87868 , 126697)** | **23658 (20370.2 , 27169.9)** | **756418 (622260 , 910774)** | **23538.3 (20220.1 , 27033.6)** | **-0.5 (-0.9 , -0.2)** |
| **Saudi Arabia** | **3185678 (2664458 , 3798700)** | **22359.2 (19209.6 , 25678.5)** | **8354742 (7033634 , 9767868)** | **21757.9 (18684.4 , 24972.1)** | **-2.7 (-7 , 2)** |
| **Sudan** | **4073440 (3420663 , 4810022)** | **23633.7 (20326.5 , 27273)** | **8893989 (7481341 , 10475764)** | **23598.8 (20291 , 27242.1)** | **-0.1 (-0.2 , -0.1)** |
| **Syrian Arab Republic** | **2568568 (2134149 , 3056543)** | **23643 (20335.7 , 27268)** | **3507816 (2996722 , 4066438)** | **23640.6 (20306.7 , 27263.7)** | **0 (-0.3 , 0.3)** |
| **Tunisia** | **1847599 (1559368 , 2166044)** | **23641.5 (20334.4 , 27268)** | **2863725 (2464872 , 3296804)** | **23657.2 (20350.7 , 27289)** | **0.1 (0 , 0.1)** |
| **Turkey** | **12106886 (10294139 , 14230375)** | **21513.7 (18480.2 , 24801.1)** | **19035787 (16409964 , 21823161)** | **21726.3 (18745.7 , 24838.8)** | **1 (-2.9 , 5.2)** |
| **United Arab Emirates** | **430881 (357367 , 514083)** | **23723.8 (20416.9 , 27245.6)** | **2512643 (2048289 , 3008501)** | **23676 (20386.9 , 27204.5)** | **-0.2 (-0.5 , 0)** |
| **Yemen** | **2569170 (2130506 , 3053770)** | **23671.1 (20362 , 27297.6)** | **6749015 (5678757 , 8023953)** | **23663 (20359.2 , 27287.1)** | **0 (-0.1 , 0.1)** |
