# Supplementary material for: Burden of tension-type headache in the Middle East and North Africa region, 1990-2019
Source: J Headache Pain. 2022 Jul 6;23(1):77. doi: 10.1186/s10194-022-01445-5 (PMC9258079; doi:10.1186/s10194-022-01445-5)
Supplement: Supplementary file 1 — Additional file 1: Table S1. Prevalence of tension-type headache in 1990 and 2019 for both sexes and the percentage change in the age-standardised rates (ASRs) per 100000 in the North Africa and the Middle East region (Generated from data available from http://ghdx.healthdata.org/gbd-results-tool). Table S2. Incidence of tension-type headache in 1990 and 2019 for both sexes and the percentage change in the age-standardised rates (ASRs) per 100000 in the Middle East and North Africa region (Generated from data available from http://ghdx.healthdata.org/gbd-results-tool). Table S3. YLDs due to tension-type headache in 1990 and 2019 for both sexes and the percentage change in the age-standardised rates (ASRs) per 100000 in the Middle East and North Africa region (Generated from data available from http://ghdx.healthdata.org/gbd-results-tool). Figure S1. The percentage change in the age-standardised point prevalence of tension-type headache in the Middle East and North Africa region from 1990 to 2019, by sex and country. (Generated from data available from http://ghdx.healthdata.org/gbd-results-tool). Figure S2. The percentage change in the age-standardised incidence of tension-type headache in the Middle East and North Africa region from 1990 to 2019, by sex and country. (Generated from data available from http://ghdx.healthdata.org/gbd-results-tool). Figure S3. The percentage change in the age-standardised YLDs of tension-type headache in the Middle East and North Africa region from 1990 to 2019, by sex and country. YLD= years lived with disability. (Generated from data available from http://ghdx.healthdata.org/gbd-results-tool). [file 10194_2022_1445_MOESM1_ESM.zip › Supplementary Table 2, Incidence, TTH, MENA.docx]

| **Table S2: Incidence of tension-type headache in 1990 and 2019 for both sexes and percentage change in age-standardised rates (ASRs) per 100,000 in the North Africa and the Middle East region**  **(Generated from data available from http://ghdx.healthdata.org/gbd-results-tool)** | | | | | |
| --- | --- | --- | --- | --- | --- |
|  | **1990** | | **2019** | | **Percentage change in ASRs per 100,000** |
|  | **No (95% UI)** | **ASRs per 100,000 (95% UI)** | **No (95% UI)** | **ASRs per 100,000 (95% UI)** |  |
| **North Africa and Middle East** | **27556747 (23868581 , 31392842)** | **8606.2 (7548.1 , 9641.1)** | **52879780 (46137009 , 59485212)** | **8680.1 (7631.6 , 9732.5)** | **0.9 (-0.1 , 1.7)** |
| **Afghanistan** | **871954 (753174 , 999212)** | **8480 (7411.2 , 9548.8)** | **2938263 (2512679 , 3388697)** | **8515.2 (7457.9 , 9593.9)** | **0.4 (0.2 , 0.6)** |
| **Algeria** | **2011748 (1729994 , 2304366)** | **8509.3 (7450.1 , 9587.6)** | **3544462 (3089897 , 4021845)** | **8501 (7440.7 , 9578.6)** | **-0.1 (-0.1 , -0.1)** |
| **Bahrain** | **42390 (36204 , 48741)** | **8538.7 (7487 , 9617.4)** | **130833 (112605 , 149916)** | **8524.4 (7476 , 9592.8)** | **-0.2 (-0.3 , 0)** |
| **Egypt** | **4549118 (3934857 , 5193201)** | **8748.6 (7699 , 9823.3)** | **8593585 (7460381 , 9681319)** | **8812.2 (7762.7 , 9893.9)** | **0.7 (-2.7 , 5)** |
| **Iran (Islamic Republic of)** | **5036952 (4381081 , 5700432)** | **9343.8 (8302.9 , 10437.3)** | **8430126 (7411247 , 9501060)** | **9837.5 (8687.5 , 11030.7)** | **5.3 (1.9 , 7.9)** |
| **Iraq** | **1341870 (1147836 , 1542801)** | **8508.6 (7450 , 9585.6)** | **3527971 (3048007 , 4011221)** | **8507.1 (7448.5 , 9584.3)** | **0 (0 , 0)** |
| **Jordan** | **293218 (250062 , 337233)** | **8500.6 (7445.4 , 9580.2)** | **982482 (849563 , 1113702)** | **8508.4 (7455 , 9588.3)** | **0.1 (0 , 0.2)** |
| **Kuwait** | **147440 (126196 , 168962)** | **8534.2 (7467.7 , 9559.1)** | **391309 (340667 , 446614)** | **8487.2 (7489.2 , 9540.2)** | **-0.6 (-3.3 , 2.4)** |
| **Lebanon** | **260091 (225186 , 295226)** | **8490 (7428.6 , 9567.4)** | **441957 (387091 , 499441)** | **8503.8 (7444.7 , 9581.2)** | **0.2 (0.1 , 0.3)** |
| **Libya** | **329764 (282454 , 378014)** | **8514.6 (7459 , 9592.6)** | **599202 (520188 , 678679)** | **8508.4 (7450.6 , 9585.8)** | **-0.1 (-0.2 , 0.1)** |
| **Morocco** | **2038667 (1760516 , 2329451)** | **8503.7 (7440.6 , 9578.6)** | **3107802 (2711391 , 3502989)** | **8502.5 (7441.7 , 9579.2)** | **0 (0 , 0)** |
| **Oman** | **152696 (130397 , 175384)** | **8534.7 (7488.3 , 9613)** | **403518 (342668 , 467602)** | **8531.4 (7483.3 , 9610.9)** | **0 (-0.2 , 0.1)** |
| **Palestine** | **153747 (131547 , 176807)** | **8511 (7451.3 , 9586.3)** | **405495 (349922 , 462326)** | **8503.8 (7444.3 , 9580.2)** | **-0.1 (-0.2 , 0)** |
| **Qatar** | **37926 (32188 , 43848)** | **8516.4 (7472.7 , 9601.9)** | **263229 (222394 , 306753)** | **8485.2 (7444.1 , 9569.2)** | **-0.4 (-0.7 , -0.1)** |
| **Saudi Arabia** | **1229865 (1057028 , 1407954)** | **8292.2 (7287.5 , 9333.3)** | **3081911 (2653414 , 3512212)** | **8206.1 (7168.6 , 9215.6)** | **-1 (-4 , 2)** |
| **Sudan** | **1537047 (1317291 , 1762667)** | **8494.1 (7430.5 , 9569.4)** | **3301315 (2843973 , 3772470)** | **8482.4 (7418.8 , 9558.5)** | **-0.1 (-0.2 , -0.1)** |
| **Syrian Arab Republic** | **982465 (841229 , 1130436)** | **8500.1 (7439.9 , 9577.2)** | **1259310 (1101071 , 1426066)** | **8488 (7422.9 , 9561.3)** | **-0.1 (-0.3 , 0.1)** |
| **Tunisia** | **684655 (592376 , 779431)** | **8499.7 (7438.4 , 9575.5)** | **1013213 (890081 , 1140492)** | **8502 (7441.4 , 9578.4)** | **0 (0 , 0.1)** |
| **Turkey** | **4678725 (4044386 , 5320065)** | **8136.8 (7158.4 , 9166.6)** | **7018674 (6183837 , 7932907)** | **8197.3 (7199.6 , 9252.4)** | **0.7 (-2.5 , 3.6)** |
| **United Arab Emirates** | **156589 (132552 , 180443)** | **8533.3 (7488.6 , 9618.9)** | **863143 (722148 , 1015406)** | **8528.1 (7490.2 , 9612.7)** | **-0.1 (-0.3 , 0.1)** |
| **Yemen** | **1001287 (851521 , 1168231)** | **8501.4 (7441.9 , 9577.3)** | **2528256 (2163210 , 2899036)** | **8504.5 (7443.4 , 9580.9)** | **0 (0 , 0.1)** |
