# Supplementary material for: Burden of tension-type headache in the Middle East and North Africa region, 1990-2019
Source: J Headache Pain. 2022 Jul 6;23(1):77. doi: 10.1186/s10194-022-01445-5 (PMC9258079; doi:10.1186/s10194-022-01445-5)
Supplement: Supplementary file 1 — Additional file 1: Table S1. Prevalence of tension-type headache in 1990 and 2019 for both sexes and the percentage change in the age-standardised rates (ASRs) per 100000 in the North Africa and the Middle East region (Generated from data available from http://ghdx.healthdata.org/gbd-results-tool). Table S2. Incidence of tension-type headache in 1990 and 2019 for both sexes and the percentage change in the age-standardised rates (ASRs) per 100000 in the Middle East and North Africa region (Generated from data available from http://ghdx.healthdata.org/gbd-results-tool). Table S3. YLDs due to tension-type headache in 1990 and 2019 for both sexes and the percentage change in the age-standardised rates (ASRs) per 100000 in the Middle East and North Africa region (Generated from data available from http://ghdx.healthdata.org/gbd-results-tool). Figure S1. The percentage change in the age-standardised point prevalence of tension-type headache in the Middle East and North Africa region from 1990 to 2019, by sex and country. (Generated from data available from http://ghdx.healthdata.org/gbd-results-tool). Figure S2. The percentage change in the age-standardised incidence of tension-type headache in the Middle East and North Africa region from 1990 to 2019, by sex and country. (Generated from data available from http://ghdx.healthdata.org/gbd-results-tool). Figure S3. The percentage change in the age-standardised YLDs of tension-type headache in the Middle East and North Africa region from 1990 to 2019, by sex and country. YLD= years lived with disability. (Generated from data available from http://ghdx.healthdata.org/gbd-results-tool). [file 10194_2022_1445_MOESM1_ESM.zip › Supplementary Table 3, YLDs, TTH, MENA.docx]

| **Table S3: YLDs due to tension-type headache in 1990 and 2019 for both sexes and percentage change in age-standardised rates (ASRs) per 100,000 in the North Africa and the Middle East region**  **(Generated from data available from http://ghdx.healthdata.org/gbd-results-tool)** | | | | | |
| --- | --- | --- | --- | --- | --- |
|  | **1990** | | **2019** | | **Percentage change in ASRs per 100,000** |
|  | **No (95% UI)** | **ASRs per 100,000 (95% UI)** | **No (95% UI)** | **ASRs per 100,000 (95% UI)** |  |
| **North Africa and Middle East** | **193331 (63493 , 553901)** | **67.5 (23.4 , 186.7)** | **416595 (138258 , 1196825)** | **68.1 (22.8 , 195.5)** | **1 (-9.5 , 8.7)** |
| **Afghanistan** | **5910 (1946 , 17343)** | **65.2 (22.4 , 181.7)** | **19586 (6272 , 57731)** | **65.2 (22.1 , 183.3)** | **-0.1 (-9.6 , 7.9)** |
| **Algeria** | **13771 (4441 , 40154)** | **66.5 (22.9 , 184.7)** | **28460 (9650 , 79524)** | **66.7 (22.6 , 189)** | **0.3 (-10.2 , 7.9)** |
| **Bahrain** | **315 (105 , 906)** | **64.8 (21.8 , 185.5)** | **1117 (386 , 3084)** | **64.6 (21.3 , 189.3)** | **-0.3 (-10.7 , 7.7)** |
| **Egypt** | **32460 (10534 , 96789)** | **68.1 (23.4 , 195)** | **65173 (20989 , 196744)** | **69.1 (22.6 , 207.8)** | **1.4 (-10.1 , 10.8)** |
| **Iran (Islamic Republic of)** | **34437 (10919 , 102619)** | **73.7 (24.8 , 211.5)** | **70182 (23015 , 207136)** | **77.6 (24.9 , 236.4)** | **5.2 (-5.7 , 15.9)** |
| **Iraq** | **8979 (2907 , 26707)** | **65.8 (22.1 , 184.1)** | **26343 (8663 , 77009)** | **66.2 (22.1 , 190.3)** | **0.7 (-11.1 , 9.1)** |
| **Jordan** | **1967 (627 , 5948)** | **66.1 (22.3 , 185.8)** | **7406 (2514 , 21769)** | **66.2 (22.7 , 191.4)** | **0.1 (-10.9 , 8.3)** |
| **Kuwait** | **1053 (342 , 3134)** | **61.9 (19.9 , 182.4)** | **3205 (1084 , 8967)** | **62.8 (20.8 , 186.4)** | **1.5 (-10.4 , 11.9)** |
| **Lebanon** | **1913 (646 , 5455)** | **66.4 (23.1 , 187)** | **3565 (1217 , 10006)** | **67 (22.7 , 191.7)** | **0.8 (-10.8 , 9.4)** |
| **Libya** | **2213 (708 , 6669)** | **65.7 (22.2 , 183.3)** | **4920 (1694 , 13899)** | **66.4 (22.8 , 191)** | **1.1 (-11.2 , 9.5)** |
| **Morocco** | **14552 (4827 , 41924)** | **66.4 (22.9 , 185.4)** | **24818 (8463 , 69426)** | **66.5 (22.7 , 187.3)** | **0.2 (-9.8 , 8.1)** |
| **Oman** | **1037 (337 , 3115)** | **64.3 (21.6 , 181)** | **3196 (1042 , 8985)** | **64.3 (21.6 , 190.1)** | **0 (-15.4 , 10.8)** |
| **Palestine** | **1014 (325 , 3041)** | **66.4 (22.7 , 185.4)** | **2914 (952 , 8613)** | **66.2 (22.1 , 187.5)** | **-0.3 (-13 , 9.3)** |
| **Qatar** | **287 (92 , 830)** | **62.9 (20.8 , 182.6)** | **2135 (699 , 6004)** | **62 (20.7 , 184.7)** | **-1.4 (-11.6 , 7.2)** |
| **Saudi Arabia** | **8472 (2745 , 24948)** | **63.2 (21.6 , 176.5)** | **25584 (9050 , 70083)** | **63 (22 , 180.3)** | **-0.3 (-18.6 , 10.8)** |
| **Sudan** | **10504 (3394 , 30933)** | **66.1 (22.4 , 186.5)** | **23633 (7813 , 69143)** | **66.4 (23.2 , 189)** | **0.5 (-11.8 , 9.8)** |
| **Syrian Arab Republic** | **6488 (2090 , 19279)** | **66.2 (22.3 , 186.7)** | **9810 (3361 , 28381)** | **67 (22.9 , 189.7)** | **1.1 (-10.5 , 9.5)** |
| **Tunisia** | **4933 (1650 , 14398)** | **66.6 (23 , 185.2)** | **8414 (2880 , 23528)** | **67 (22.6 , 192.5)** | **0.7 (-10.7 , 8.5)** |
| **Turkey** | **35373 (12383 , 95725)** | **66 (24 , 176.6)** | **60666 (21747 , 160117)** | **66.6 (23.6 , 180)** | **0.8 (-10 , 10.1)** |
| **United Arab Emirates** | **1156 (369 , 3318)** | **63.2 (20.9 , 183.6)** | **7426 (2396 , 20517)** | **63.2 (20.7 , 187.8)** | **0 (-12.6 , 8.9)** |
| **Yemen** | **6366 (1989 , 19611)** | **65.7 (22 , 185.7)** | **17617 (5766 , 51725)** | **66.1 (22.8 , 185.6)** | **0.6 (-11.1 , 9.8)** |
