# Supplementary figures and images for: Burden of tension-type headache in the Middle East and North Africa region, 1990-2019
Source: J Headache Pain. 2022 Jul 6;23(1):77. doi: 10.1186/s10194-022-01445-5 (PMC9258079; doi:10.1186/s10194-022-01445-5)

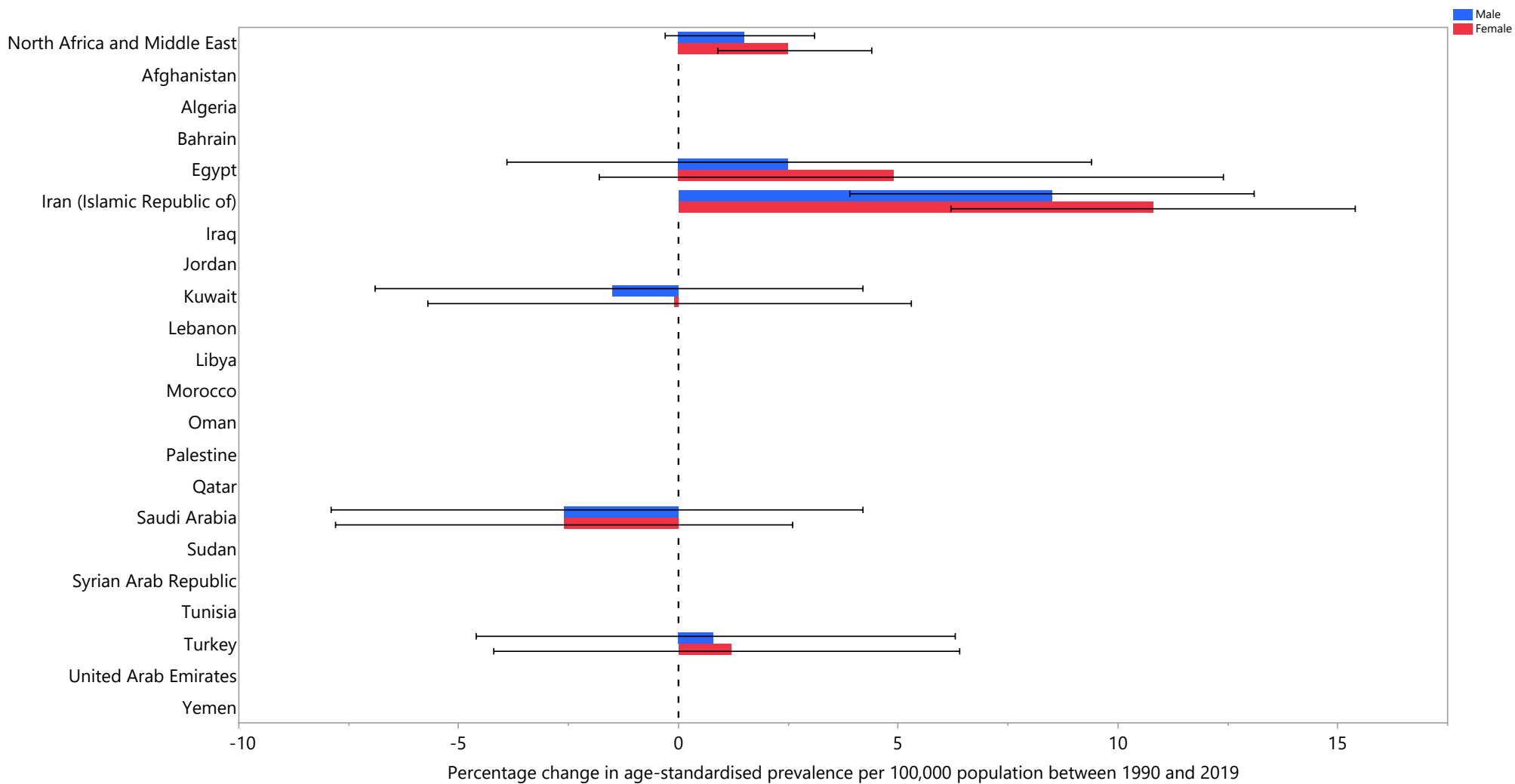

Supplement: Supplementary file 1 — Additional file 1: Table S1. Prevalence of tension-type headache in 1990 and 2019 for both sexes and the percentage change in the age-standardised rates (ASRs) per 100000 in the North Africa and the Middle East region (Generated from data available from http://ghdx.healthdata.org/gbd-results-tool). Table S2. Incidence of tension-type headache in 1990 and 2019 for both sexes and the percentage change in the age-standardised rates (ASRs) per 100000 in the Middle East and North Africa region (Generated from data available from http://ghdx.healthdata.org/gbd-results-tool). Table S3. YLDs due to tension-type headache in 1990 and 2019 for both sexes and the percentage change in the age-standardised rates (ASRs) per 100000 in the Middle East and North Africa region (Generated from data available from http://ghdx.healthdata.org/gbd-results-tool). Figure S1. The percentage change in the age-standardised point prevalence of tension-type headache in the Middle East and North Africa region from 1990 to 2019, by sex and country. (Generated from data available from http://ghdx.healthdata.org/gbd-results-tool). Figure S2. The percentage change in the age-standardised incidence of tension-type headache in the Middle East and North Africa region from 1990 to 2019, by sex and country. (Generated from data available from http://ghdx.healthdata.org/gbd-results-tool). Figure S3. The percentage change in the age-standardised YLDs of tension-type headache in the Middle East and North Africa region from 1990 to 2019, by sex and country. YLD= years lived with disability. (Generated from data available from http://ghdx.healthdata.org/gbd-results-tool). [file 10194_2022_1445_MOESM1_ESM.zip › Figure S1, Prevalence Change by Sex TTH MENA.pdf]

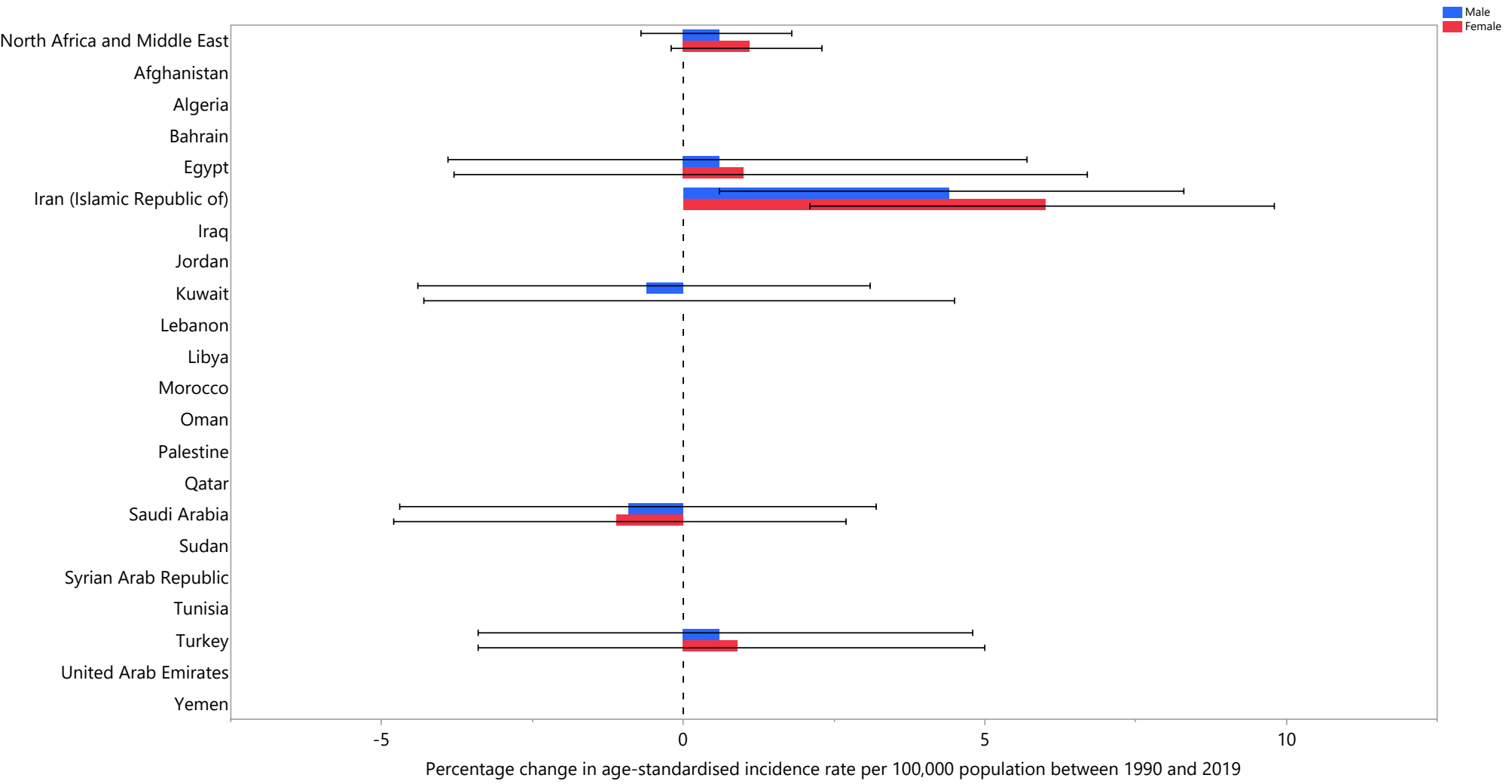

Supplement: Supplementary file 1 — Additional file 1: Table S1. Prevalence of tension-type headache in 1990 and 2019 for both sexes and the percentage change in the age-standardised rates (ASRs) per 100000 in the North Africa and the Middle East region (Generated from data available from http://ghdx.healthdata.org/gbd-results-tool). Table S2. Incidence of tension-type headache in 1990 and 2019 for both sexes and the percentage change in the age-standardised rates (ASRs) per 100000 in the Middle East and North Africa region (Generated from data available from http://ghdx.healthdata.org/gbd-results-tool). Table S3. YLDs due to tension-type headache in 1990 and 2019 for both sexes and the percentage change in the age-standardised rates (ASRs) per 100000 in the Middle East and North Africa region (Generated from data available from http://ghdx.healthdata.org/gbd-results-tool). Figure S1. The percentage change in the age-standardised point prevalence of tension-type headache in the Middle East and North Africa region from 1990 to 2019, by sex and country. (Generated from data available from http://ghdx.healthdata.org/gbd-results-tool). Figure S2. The percentage change in the age-standardised incidence of tension-type headache in the Middle East and North Africa region from 1990 to 2019, by sex and country. (Generated from data available from http://ghdx.healthdata.org/gbd-results-tool). Figure S3. The percentage change in the age-standardised YLDs of tension-type headache in the Middle East and North Africa region from 1990 to 2019, by sex and country. YLD= years lived with disability. (Generated from data available from http://ghdx.healthdata.org/gbd-results-tool). [file 10194_2022_1445_MOESM1_ESM.zip › Figure S2, Incidence Change by Sex TTH MENA.pdf]

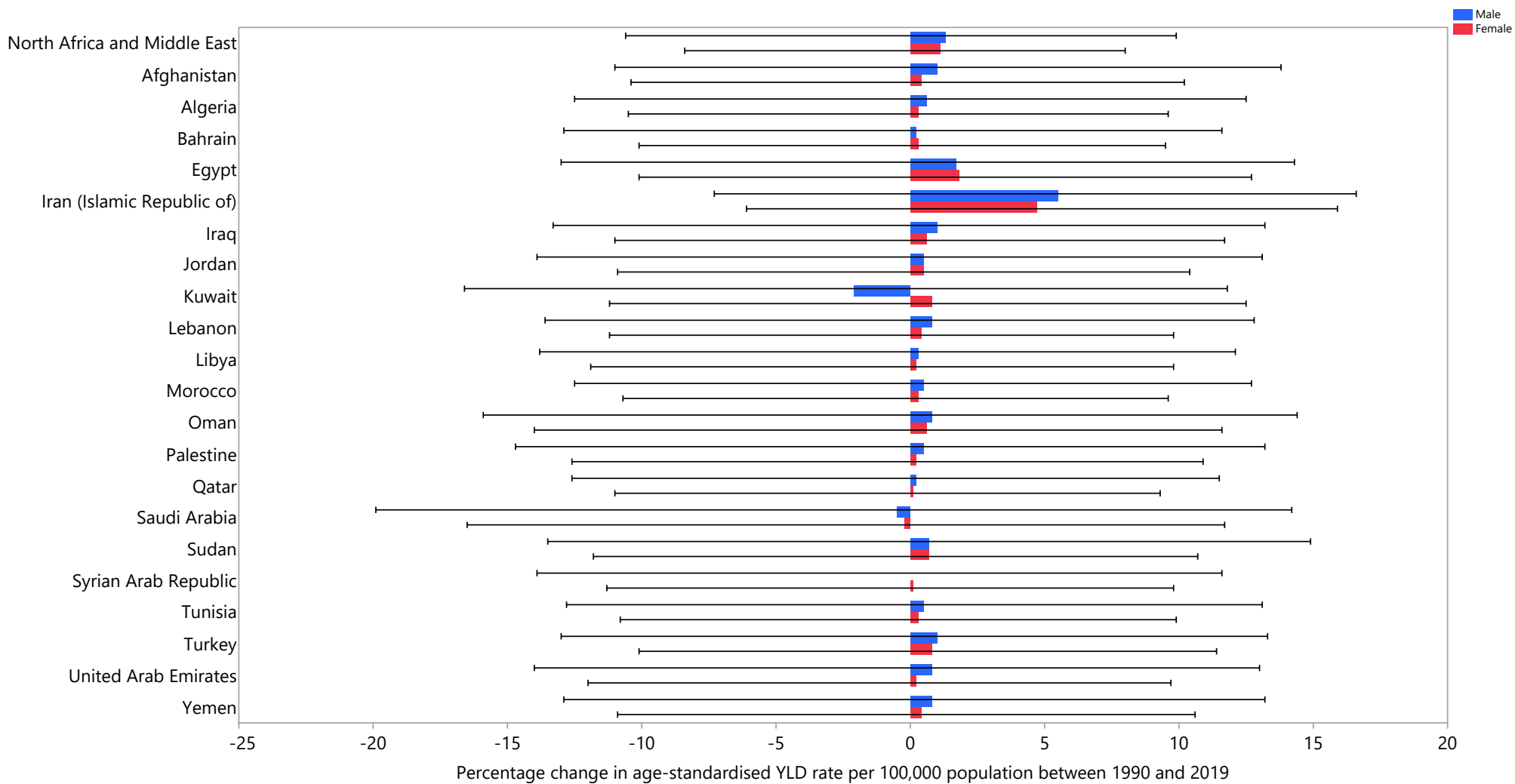

Supplement: Supplementary file 1 — Additional file 1: Table S1. Prevalence of tension-type headache in 1990 and 2019 for both sexes and the percentage change in the age-standardised rates (ASRs) per 100000 in the North Africa and the Middle East region (Generated from data available from http://ghdx.healthdata.org/gbd-results-tool). Table S2. Incidence of tension-type headache in 1990 and 2019 for both sexes and the percentage change in the age-standardised rates (ASRs) per 100000 in the Middle East and North Africa region (Generated from data available from http://ghdx.healthdata.org/gbd-results-tool). Table S3. YLDs due to tension-type headache in 1990 and 2019 for both sexes and the percentage change in the age-standardised rates (ASRs) per 100000 in the Middle East and North Africa region (Generated from data available from http://ghdx.healthdata.org/gbd-results-tool). Figure S1. The percentage change in the age-standardised point prevalence of tension-type headache in the Middle East and North Africa region from 1990 to 2019, by sex and country. (Generated from data available from http://ghdx.healthdata.org/gbd-results-tool). Figure S2. The percentage change in the age-standardised incidence of tension-type headache in the Middle East and North Africa region from 1990 to 2019, by sex and country. (Generated from data available from http://ghdx.healthdata.org/gbd-results-tool). Figure S3. The percentage change in the age-standardised YLDs of tension-type headache in the Middle East and North Africa region from 1990 to 2019, by sex and country. YLD= years lived with disability. (Generated from data available from http://ghdx.healthdata.org/gbd-results-tool). [file 10194_2022_1445_MOESM1_ESM.zip › Figure S3, YLD Change by Sex TTH MENA.pdf]
